# Quality of life in adolescents with idiopathic scoliosis: A cross-sectional comparison

**DOI:** 10.4102/sajp.v81i1.2099

**Published:** 2025-08-14

**Authors:** Kathrin Güttinger, Cornelia Neuhaus, Ariane Schwank

**Affiliations:** 1 Department of Physiotherapy, Faculty of Health, Zurich University of Applied Sciences, Winterthur, Switzerland; 2 Skoliopraxis, Winterthur, Switzerland; 3 Institute for Therapy and Rehabilitation, Kantonsspital Winterthur, Winterthur, Switzerland; 4 Department of Physiotherapy, Faculty of Therapy, University Children’s Hospital Basel, Basel, Switzerland; 5 MOVANT Research Group, Department of Rehabilitation Sciences and Physiotherapy, University of Antwerp Wilrijk, Antwerp, Belgium

**Keywords:** adolescents, scoliosis, quality of life, KIDSCREEN-27, mental health, physiotherapy

## Abstract

**Background:**

Idiopathic scoliosis is a three-dimensional spinal curvature treated during adolescence with physiotherapy, braces or surgery. This can be stressful for patients. Few studies compare the quality of life of adolescents with and without scoliosis. Our study aims to investigate the quality of life of adolescents with and without scoliosis using the KIDSCREEN-27 questionnaire.

**Objectives:**

The aim of this study was to investigate the quality of life of adolescents with scoliosis in comparison to adolescents without scoliosis.

**Method:**

This comparative cross-sectional study included 60 participants who answered KIDSCREEN-27 accompanied by questions about age, sport intensity and their school category. Girls between the ages of 12 and 17 were included in the study. For each category of KIDSCREEN-27, the values were compared between participants with and without scoliosis.

**Results:**

Statistical analysis was done using R Version 4.3.3. In none of the categories were the mean *T*-scores of adolescents with scoliosis significantly lower than those of the comparison group. In the scoliosis group, 33% (*n* = 10) showed values rated as ‘low’ quality of life in the category ‘physical well-being’. In the category ‘psychological well-being’, 36% (*n* = 11) showed ‘low’ values.

**Conclusion:**

A general statement about the quality of life in adolescents with scoliosis cannot be made, but monitoring during treatment appears to be important. It is a complex construct that varies for each patient. KIDSCREEN-27 can quickly and easily identify low quality of life in patients with scoliosis.

**Clinical implications:**

Physiotherapists play an important role in the scoliosis treatment team as they usually see the patients most often. Thus, early recognition of impaired quality of life is crucial to offer a targeted therapy plan.

## Introduction

Scoliosis is a three-dimensional deformity of the spine and trunk that typically manifests during adolescence (Rigo & Grivas 2010). Because of the risk of progression during the growth phase, scoliosis should be treated to prevent worsening. Treatment options include physiotherapy, bracing or surgical intervention. The Cobb angle, measured on X-rays, is the gold standard for assessing scoliosis (Cassar-Pullicino & Eisenstein 2002) and is mainly used to define ‘successful treatment outcome’ (Joarder et al. 2023). Additionally, it is important to assess patient-related outcomes regarding the quality of life (QoL) (Ceballos-Laita et al. 2023). Study designs and methodologies for evaluating QoL in adolescents with scoliosis differ greatly.

Some research findings indicate a reduced QoL in adolescents with scoliosis compared to healthy adolescents or norm data, measured with a generic questionnaire (Torén & Diarbakerli 2022). Conversely, some studies show higher QoL scores in adolescents with scoliosis (Kontodimopoulos et al. 2018; Ugwonali et al. 2004). This may be related to the fact that the participants were newly diagnosed (Kontodimopoulos et al. 2018) and that the parents, rather than the children, were surveyed (Ugwonali et al. 2004).

Numerous studies described that adolescents with scoliosis often face psychosocial challenges during treatment, such as decreased self-esteem, deteriorating mental health and increased stress levels (Kaya et al. 2022; Wang et al. 2021; Zimoń et al. 2018). The outcome measures are mostly scoliosis-specific questionnaires, and most of the study designs lack a comparison to a healthy comparison group. Predominantly, the Scoliosis Research Society Score (SRS) 22 questionnaire measures QoL in patients with scoliosis (Aulisa et al. 2010; Negrini et al. 2018), but this does not allow direct comparisons with healthy individuals. However, the most significant impact of illness on QoL is observed when comparing individuals with the condition to healthy, asymptomatic individuals (Ravens-Sieberer 2006). Furthermore, the increasing recognition of the unique needs of children has led to a growing emphasis on QoL measurement questionnaires designed for children and adolescents rather than adults (Ravens-Sieberer 2006). Despite this, few studies have employed generic QoL questionnaires specifically designed for adolescents to assess adolescents with scoliosis (Kontodimopoulos et al. 2018; Schwieger et al. 2016).

This study aimed to enhance understanding of the impact of scoliosis on QoL in adolescents. The research question was:

Is there a difference in perceived QoL, obtained by the questionnaire KIDSCREEN-27 and one supplementing question, ‘Self-Perception’ from KIDSCREEN-52, in adolescents with scoliosis compared to adolescents without scoliosis?

## Research methods and design

### Study design

A cross-sectional comparative design was used for this study.

#### Setting, study population and sampling strategy

Adolescents with scoliosis from four private physiotherapy practices and three hospitals in the area of Zurich were invited to participate. Adolescents in the hospital were invited to participate via letter, while those in physiotherapy private practices were personally approached by their therapists.

The comparison group contained learners from four different schools surrounding Zurich. Their teachers personally approached them.

Initially, the participants received explanations about our study in writing (for those contacted by letter) or both in writing and verbally (for those approached by their therapists or teachers).

Given that scoliosis treatment is particularly crucial when the spine is still growing, participants aged 12 to 18 years were included in the study.

The inclusion criteria for the participants in the scoliosis group were diagnosis of idiopathic scoliosis, female and Cobb angle > 25°. The exclusion criteria were secondary scoliosis and male or female without scoliosis or with other illnesses.

The inclusion criteria for the comparison group were female. The exclusion criteria for the comparison group were male and female, diagnosed with scoliosis or other illnesses.

Quality of life in adolescents between the ages of 12 and 17 can be different, so care was taken to ensure that the groups were very similar in age. Male adolescents were excluded because the QoL of boys differs from girls (Aulisa et al. 2010; Zimoń et al. 2018). Additionally, scoliosis is much more prevalent in girls (Rainoldi et al. 2015). Because sports can influence QoL, participants were asked about their level of physical activity to account for potential confounding bias related to sports.

Power analysis indicated that 23 participants were needed in each group for statistical significance at 90% power and alpha 0.05. This is based on an estimated standard deviation (s.d.) of 8 points for each group and a minimum difference of 8 points (in the KIDSCREEN-27 score in every category) we intended to detect between groups (Ravens-Sieberer 2006).

The study included 60 participants, comprising 30 individuals with scoliosis and 30 healthy adolescents as a comparison group. The participants answered the questions once; there was no repeat survey at a later date.

### Data collection

All the study participants completed the KIDSCREEN-27 once and the additional category ‘Self-Perception’ from the KIDSCREEN-52, along with questions about the intensity of their physical activity and the school category or educational track they were in. Participants in the scoliosis group also answered questions regarding the wearing time of the brace, the Cobb angle and the intensity of their therapy.

The KIDSCREEN instrument was developed within the project ‘Screening and Promotion for Health-related QoL in Children and Adolescents – A European Public Health Perspective’ to evaluate and standardise the general QoL of healthy and chronically ill children and adolescents. The questionnaire aims to detect children at risk of health-related QoL restriction (Ravens-Sieberer 2006).

KIDSCREEN-27 includes the following five dimensions: ‘Physical Well-being’, ‘Psychological Well-being’, ‘Autonomy and Parent Relation’, ‘Social Support and Peers’ and ‘School Environment’. It takes 10 min to 15 min to complete the whole questionnaire. The higher the score, the better the QoL. The internal consistency was reported to be good to excellent in all the dimensions (ranging from 0.80 to 0.84) (Ravens-Sieberer 2006).

The supplementing question ‘Self-Perception’ was chosen because we hypothesise that adolescents with scoliosis could have reduced values in ‘Self-Perception’ (Rusovs et al. 2013; Wang et al. 2021).

The returned questionnaires were anonymised, and all subsequent data processing was conducted in an anonymised manner.

### Data analysis

For all continuous data, the mean, the median and the s.d. were calculated for the group of adolescents with and without scoliosis.

Statistical analysis was done using R Version 4.3.3. Through Rasch analysis, the raw data, initially ordinal in nature, were transformed into interval-scaled *T*-values, allowing mathematical calculations. The raw data of each study participant were converted into Rasch person parameters *P*, and then the KIDSCREEN *T*-values were calculated using the following formula (Equation 1):


T=(P−μ/σ)×10+50 [Eqn 1]

μ = mean person parameter of the standard population, σ = standard deviation of the person parameters in the standard population.

The person parameter *P* for each subcategory of KIDSCREEN, as well as μ and σ, was taken from the KIDSCREEN manual. A value ± ½ s.d. of the mean *T*-values of the norm data can be defined as ‘high or low’ QoL. The mean ± ½ s.d. range is categorised as the ‘normal’ range (Ravens-Sieberer 2006).

The a priori hypothesis: H0: *µ1 = µ2, H1: µ1 ≠µ2*, was analysed with the t-test between the groups with or without scoliosis.

In the scoliosis group, Spearman’s correlation coefficient was calculated between the *T*-scores and the data about Cobb angle, age, brace, therapy duration, sport and school. A correlation was classified as significant when *p* < 0.05 (Doi et al. 2021).

### Ethical considerations

The Ethics Committee of Zurich (Switzerland) accepted the study (Ref. Nr. KEK-ZH-Nr. 2013-0402). The caregivers and the participants provided written informed consent before the start of the study. All adolescents participated voluntarily.

## Results

### Participants

To collect questionnaires from 30 adolescents with scoliosis, we invited 61 individuals. Similarly, to achieve 30 responses from adolescents without scoliosis, we approached 85 individuals to participate in our study.

The study included 60 participants, comprising 30 individuals with scoliosis and 30 healthy adolescents as a comparison group.

### Descriptive data

Table 1 presents descriptive data from the two study groups. The complete data frame is available on request.

**TABLE 1:** Descriptive characteristics of adolescents with and without scoliosis.

Characteristics	Adolescents with scoliosis *n* = 30					Control group *n* = 30				
	Mean	s.d.	Range	*n*	%	Mean	s.d.	Range	*n*	%
Age (years)	14	1.8	12–17	-	-	14	1.0	12–17	-	-
Sport 3x/week or more	-	-	-	12	40	-	-	-	11	36
Sport 1–2x/week	-	-	-	11	36	-	-	-	16	53
No sport	-	-	-	7	23	-	-	-	3	10
Cobb angle	36.1°	10	25°–55°	-	-	n/a	n/a	n/a	n/a	n/a
Physical therapy month	30.1	26.4	26.4	-	-	n/a	n/a	n/a	n/a	n/a
Number of braced patients	-	-	-	8	26	n/a	n/a	n/a	n/a	n/a

s.d., standard deviation; n/a, not applicable.

### Main results

In none of the categories were the mean *T*-scores of adolescents with scoliosis significantly lower than those of the comparison group or the norm data of Swiss girls (see Table 2) (Ravens-Sieberer 2006). In the comparison group, the mean of the category ‘Self-Perception’ was just below the range of ‘normal’ *T*-scores and therefore defined as low. In this category, the mean *T*-score of adolescents without scoliosis was lower than that of adolescents with scoliosis.

**TABLE 2:** Mean *T*-scores in all categories of the KIDSCREEN-27.

Categories	*T*-scores							Median		95% CI§
	Norm Switzerland†		Range of ‘normal’ scores‡	Adolescents with scoliosis		Control group		Adolescents with scoliosis	Control group	
	Mean	s.d.		Mean	s.d.	Mean *T*-scores	s.d.			
‘Physical Well-being’	49.9	8.4	45.7 to 54.1	52.0	9.1	51.9	9.4	52.4	52.4	−4.8 to 4.7
‘Psychological Well-being’	50.2	8.8	45.8 to 54.6	48.9	7.4	46.1	8.4	47.5	45.7	−6.9 to 1.3
‘Autonomy and Parent relation’	52.1	8.4	47.8 to 56.3	52.0	7.0	55.0	9	51.2	53.3	−1.2 to 7.1
‘Social Support and Peers’	51.2	8.5	46.9 to 55.4	51.1	7.7	53.1	7.9	49.8	53.2	−2.2 to 5.9
‘School Environment’	50.8	7.9	46.8 to 54.8	52.8	8.0	51.5	6.8	52.7	51.07	−5.2 to 2.5
‘Self-Perception’	48.3	8.6	44.0 to 52.6	47.6	7.6	43.4	6.2	46.9	43.9	−7.9 to -0.6

s.d., standard deviation.

† , Ravens-Sieberer, U., 2006, *The KIDSCREEN questionnaires - Quality of life questionnaires for children and adolescents*, Pabst Science Publishers, Lengerich;

‡ , Mean *T*- scores Norm Switzerland ± ½ s.d.;

§ , difference of the mean of the *T*-values between the control group and adolescents with scoliosis.

### Other analyses

#### ‘Low’, ‘normal’ and ‘high’ quality of life

In the scoliosis group, 6% (*n* = 2) of the adolescents had ‘low’ *T*-scores in all categories. In the comparison group, no adolescent had ‘low’ scores in all categories. In the scoliosis group and in the comparison group, 30% (*n* = 9 in each group) of the adolescents scored ‘low’ in three or more categories. In the scoliosis group, 10% (*n* = 3) of the adolescents scored ‘high’ in five or six categories. No healthy adolescent reached a ‘high’ score in five or more categories. The details for all adolescents about ‘low’, ‘normal’ and ‘high’ QoL are given in Online Appendix 1, Table 1-A1.

A total of 33% (*n* = 10) of the participants in the scoliosis group scored ‘low’ in ‘Physical Well-being’ and/or ‘Psychological Well-being’. The full data about how many adolescents score ‘low’, ‘normal’ or ‘high’ are provided in Online Appendix 1, Table 2-A1.

#### Correlation analysis

In the patient group, the correlations between the mean *T*-scores of the categories and the Cobb angle, the age, the duration of therapy (months), the brace-wearing time (h/day), the sport intensity (h/week) or the school type were not significant (*p* < 0.05).

#### Mean *T*-scores in subgroups

In Table 3, the mean *T*-scores for the categories are presented separately for two groups: Patients with a Cobb angle ≥ 45° and with a Cobb angle < 45° (Parent et al. 2010).

**TABLE 3:** *T*-scores in subgroups < and ≥ 45° Cobb angle and *t*-test between groups.

Categories	*T*-scores of adolescents with scoliosis				95% CI†	*p*	Cohens *d*
	Cobb < 45° (*n* = 22)		Cobb ≥ 45° (*n* = 8)				
	Mean	s.d.	Mean	s.d.			
‘Physical Well-being’	53	9.3	47	6.8	−12.8 – 0.4	0.07	−0.70
‘Psychological Well-being’	50	7.7	44	4.3	−10.8 – -1.5	0.01	−0.88
‘Autonomy and Parent relation’	53	7.6	49	4.1	−7.9 – 1.1	0.13	−0.49
‘Social Support and Peers’	52	7.3	46	7.3	−12.8 – 0.3	0.06	−0.85
‘School Environment’	54	7.5	48	8.4	−12.9 – 1.8	0.13	−0.72
‘Self-Perception’	48	7.8	44	6.2	−10.8 – 0.9	0.09	−0.66

s.d., standard deviation; CI, confidence interval.

† , 95% CI difference of the mean of the *T*-values between groups.

In the category ‘Psychological Well-being’, the adolescents with a Cobb angle *≥* 45° scored notably lower in *T*-scores than adolescents with scoliosis < 45° Cobb angle (*p* = 0.01).

## Discussion

This study investigated whether the QoL, based on KIDSCREEN-27 and one extra question about ‘Self-Perception’, in adolescents with scoliosis was lower than the QoL in adolescents without scoliosis.

### Key findings

Against our hypothesis, the female adolescents with scoliosis reached very similar QoL mean values in comparison to our healthy comparison group or to the norm data.

In the category ‘Psychological Well-being’, the adolescents with a Cobb angle ≥ 45° scored notably lower in *T*-scores for QoL than patients with a Cobb angle < 45°.

33% (*n* = 10) of the adolescents with scoliosis had reduced QoL in the categories ‘Psychological Well-being’ and/or ‘Physical Well-being’. Six per cent (*n* = 2) of the adolescents with scoliosis had ‘low’ *T*-scores in all categories. In the comparison group, no adolescent had ‘low’ scores in all categories.

### Discussion of key findings

It is widely recognised that scoliosis is a risk factor for reduced QoL (Kaya et al. 2022; Torén & Diarbakerli 2022). This is contrary to our findings. We recognised participants with scoliosis with reduced QoL, but in general, the adolescents with scoliosis did not have lower scores than the healthy comparison group. There are few studies that have also obtained such results. One study detected even better results in almost every category of KIDSCREEN-52 in participants in the scoliosis group. They included only newly diagnosed patients; therefore, it is unknown whether the QoL would have changed after treatment began (Kontodimopoulos et al. 2018). In a study of 1205 learners with scoliosis, the deformity did not impact the adolescents greatly until a diagnosis was made and treatment was planned or contact with specialists established (Rainoldi et al. 2015). In most studies, QoL is observed during treatment. Therefore, it is difficult to determine whether the reduced QoL is because of scoliosis or the treatment.

The QoL in adolescents with scoliosis is mostly measured with scoliosis-specific questionnaires. However, a generic questionnaire like KIDSCREEN may be insufficient for detecting a reduced QoL in patients with scoliosis. Kontodimopoulos et al. (2018) combined KIDSCREEN-52 with SRS 22 (very often used to measure QoL in patients with scoliosis) for their measures of QoL in patients with scoliosis. In their analysis, the category ‘Physical Well-being’ (identical to the category in KIDSCREEN-27) was a significant predictor for all four dimensions in the SRS 22 Questionnaire. The authors concluded that the generic instrument KIDSCREEN-52 is detecting disease-specific QoL aspects (Kontodimopoulos 2018).

It seems like the QoL of the patients with scoliosis is influenced by more complex constructs than scoliosis.

### What influences quality of life in patients with scoliosis?

A large body of research acknowledges influencing factors for QoL in adolescents with scoliosis (Anwer et al. 2015; Kaya et al. 2022; Parent et al. 2010; Salah et al. 2023; Schreiber et al. 2013; Tavernaro et al. 2012; Wang et al. 2021), which seems important when studying this construct. In our study, we additionally performed correlation analysis between the categories of KIDSCREEN 27 and the category ‘Self-Perception’ from KIDSCREEN-52 and different characteristics around the individual patient to seek more insight into our data.

The most mentioned associations to low QoL in conservative treatment were the brace treatment and the Cobb angle (Salah et al. 2023; Wang et al. 2021). Our results may not support the finding that brace treatment correlated to QoL, as only 8 (26%) participants in the scoliosis group wore a brace, which is an insufficient number to detect a true correlation. But other findings strengthen this relationship. Ugwonali et al. (2004) compared 78 braced patients to 136 non-braced in the comparison group. They showed the surprising result that the QoL of the braced patients was not below the not braced or the norm data. There the QoL was measured by evaluation of the parents’ perspective, which might have biased the degree of QoL and hindered a true comparison. Further research found that the QoL of adolescents with scoliosis treated with a brace was similar to the adolescents with scoliosis without a brace (observation treatment) or to healthy children. They found decreased QoL in patients who did not participate in their treatment decisions, regardless of whether they were undergoing brace or observation treatment (Schwieger et al. 2016).

Quality of life during brace treatment may also be influenced by the Cobb angle correction during treatment. Researchers hypothesised that a decreasing Cobb angle supported the patients’ feeling of experiencing an effective treatment, which reduced their stress levels of wearing a brace (Khoshhal et al. 2019). In another study, patients experienced decreasing QoL in the first period of brace treatment, but afterwards, this effect vanishes (Di Maria et al. 2023). In our study, we did not ask the braced patients at what time they started the treatment or how their Cobb angle changed. Factors that could have influenced our results remain, therefore, unknown.

We did see a difference in QoL in all KIDSCREEN 27 categories and in the category ‘Self-Perception’ from KIDSCREEN-52. We recognised a significant difference (*p* = 0.01) in ‘Psychological Well-being’ when we performed a subgrouping of the participants in the scoliosis group into one group with Cobb angles of ≥ 45° and one group with Cobb angles < 45°. The participants with Cobb angles of ≥ 45° had lower scores in the mentioned categories than the participants with smaller curves. This is in accordance with the recognised association between severity of the deformity and QoL. Quality of life remains steady until the curve reaches a critical Cobb angle of about 45 degrees (surgical threshold), beyond which QoL begins to decline linearly as deformity worsens. The relationship between QoL and scoliosis severity is more accurately represented by segmented models than by linear ones (Parent et al. 2010). The question is whether the deformity decreases the QoL or whether the recommended treatment for the deformity does. This is challenging to analyse, as guidelines suggest informing patients with a Cobb angle of 45° or more about the option for surgery. Interestingly, if patients were compliant with the treatment, they had remarkably better QoL scores than patients who were non-compliant (Rivett, Stewart & Potterton 2014). In our study, we did not ask about the initial Cobb angle and about the compliance. So, it remains unclear whether patients with Cobb angles ≥ 45° experienced deformity progression because of non-compliance. Hence, we do not know if the Cobb angle ≥ 45° was the cause or the consequence of the low scores.

Sport might be another influencing factor for QoL in patients with scoliosis. Research findings indicated that adolescents with scoliosis are less likely to participate in sports or exercises compared to healthy adolescents. And their QoL, as measured by EQ-5D-5L, was lower (Torén & Diarbakerli 2022). This is in line with a described relationship between movement and improved satisfaction in adolescents with scoliosis (Tones, Moss & Polly 2006). And with the observation that physical activity reduced mental distress in adolescents with scoliosis (Leszczewska et al. 2012). It is therefore possible that the physical activity of our patient group – which did not differ from the comparison group – had a positive influence on their QoL. As exercises in physical therapy may impact QoL positively (Anwer et al. 2015; Schreiber et al. 2013), it could have influenced the QoL of our study groups too.

Potentially, a treatment team of doctors, orthotists and physical therapists working hand in hand with the patient who undergoes a scoliosis treatment could influence the QoL as a main factor. The management of the patients, often overlooked, emerges as a pivotal determinant of QoL (Tavernaro et al. 2012). In this study, the management of the participants with scoliosis was not evaluated because the participants were from different clinics, and a treatment team like that described in the study of Tavernaro et al. (2012) did not exist.

There are many factors that can influence the complex construct of QoL in patients with scoliosis. Research indicates that QoL and supporting mental health in scoliosis patients during treatment need more attention (Wang et al. 2021). However, this is still not part of the standard assessment or therapy goals in Switzerland. We therefore recommend implementing the QoL in the standard assessment.

### Strengths and limitations

Hypothetically, our results were influenced by a selection bias because only every second participant in the scoliosis group was willing to participate, and the less compliant might have rejected their inclusion.

This study provides interesting insight into the QoL in adolescents with scoliosis but is limited in its external validity. The sample size was calculated for a comparison analysis between two groups and is too small to draw conclusions about the general scoliotic population. Especially when examining correlations with patient characteristics or factors influencing QoL, our data are too limited.

### Clinical implications

In general, it is known that good QoL in adolescents is crucial for a healthy transition to adulthood, and it is the foundation for health in life (Inchley et al. 2020; Ravens-Sieberer et al. 2012). Therefore, it seems important to be aware of the QoL in this period. To seek insight into an adolescent’s perspective during scoliosis treatment seems challenging. Adolescents with scoliosis tend to be introverted (D’Agata, Sánchez-Raya & Bagó 2017), and a high percentage of parents of adolescents with scoliosis are unaware of their children’s emotional and behavioural problems (Sanders et al. 2018). If parents struggle with this, healthcare providers might face similar challenges. On the other hand, patients with scoliosis desire more often a conversation about mental health than healthcare providers are offering (Zeck & Glahn Castille 2023). But there is a growing emphasis on recognising the child’s perspective as equally or more relevant than the one of experts or the findings of literature reviews (Ravens-Sieberer 2006).

Physical therapists play an important role in the treatment team for patients with scoliosis and often see these patients more frequently than other disciplines involved (Tavernaro et al. 2012). Thus, it is important that physical therapists know how to detect and observe reduced QoL in patients with scoliosis efficiently. If it is recognised early, physical therapists can offer possible solutions and interact with other disciplines to improve the situation for the patient.

The KIDSCREEN-27 could be a valuable tool for clinical practice, offering a practical way to assess the QoL of patients with scoliosis compared to healthy adolescents. It provides a basis for offering interventions or adapting treatment procedures to improve QoL.

Assessing QoL is important throughout the entire treatment process. However, there are times when monitoring the QoL should be emphasised, such as the beginning of brace treatment, which is a particularly challenging period for patients with scoliosis (Di Maria et al. 2023). It is also known that physical therapy and physical activity can improve QoL (Anwer et al. 2015; Leszczewska et al. 2012; Schreiber et al. 2013). So this could be highlighted during the difficult initial phase of brace treatment, or psychological support can be offered.

Quality of life is connected with compliance, and compliance is connected to treatment success of objective measurements like the Cobb angle (Rivett et al. 2014). To be able to offer patients tailored therapy, the recording of QoL in a way that can be integrated into everyday practice should be included.

### Future research

Methodologically, we suggest for future studies to integrate the questionnaire into the clinical setting where all participants with scoliosis answer the questionnaire as part of their assessment to avoid potential selection bias because of non-compliance.

Future research should seek to integrate the point of view of the adolescent patients, their mental well-being and their input about treatment success. Qualitative studies might explore these elements more deeply and could strengthen a patient-oriented treatment approach (Essex et al. 2021; Joarder et al. 2023).

## Conclusion

It was surprising that the adolescents with scoliosis did not score lower in the KIDSCREEN-27 tool than adolescents without scoliosis. Our research shows that scoliosis and its treatment during adolescents do not necessarily lead to reduced QoL. It is very complex and can be influenced by many factors. There is a tendency that a Cobb angle of ≥ 45° elicits reduced QoL. But clear evidence necessitates further investigations with larger sample sizes. This study clearly demonstrates the rapid and easy detection of low QoL in individual patients using KIDSCREEN-27, potentially vital for treatment success.

Adolescence, a crucial developmental stage, lays the foundation for adulthood, warranting careful attention and support. Finally, QoL is subjective, and it is important for clinicians and researchers to get to know this point of view of the patients. It seems that it cannot be derived from objective or clinical parameters. Quality of life should be captured for every individual patient and in the different phases of treatment.

If we ‘want to look after the person, not just the curve’ (Rigo 2024), we need to get connected with the adolescents and their subjective point of view of the situation. Osler (1849–1919), a physician, stated: ‘It is much more important to know what sort of patient has the disease than what sort of disease a patient has’ (John 2013).
